# Antiviral activity of *Acanthaster planci* phospholipase A2 against human immunodeficiency virus

**DOI:** 10.14202/vetworld.2018.824-829

**Published:** 2018-06-20

**Authors:** Anondho Wijanarko, Kenny Lischer, Heri Hermansyah, Diah Kartika Pratami, Muhamad Sahlan

**Affiliations:** 1 Department of Chemical Engineering, Faculty of Engineering, Universitas Indonesia, Indonesia; 2 Department of Pharmacognosy-Phytochemistry, Faculty of Pharmacy, Universitas Indonesia, Indonesia; 3 Research Center for Biomedical Engineering, Faculty of Engineering, Universitas Indonesia, Indonesia

**Keywords:** *Acanthaster planci*, antiviral activity, human immunodeficiency virus, Indonesia, phospholipase

## Abstract

**Aim:**

Investigation of antiviral activity of *Acanthaster planci* phospholipase A2 (AP-PLA2) from moluccas to human immunodeficiency virus (HIV).

**Materials and Methods:**

Crude venom (CV) and F20 (PLA2 with 20% fractioned by ammonium sulfate) as a sample of PLA 2 obtained from *A. planci’s* extract were used. Enzymatic activity of PLA2 was determined using the degradation of phosphatidylcholine (PC). Activity test was performed using *in vitro* method using coculture of phytohemagglutinin-stimulated peripheral blood mononuclear cell (PBMC) from a blood donor and PBMC from HIV patient. Toxicity test of AP-PLA2 was done using lethal concentration required to kill 50% of the population (LC_50_).

**Results:**

AP-PLA2 F20 had activity and purity by 15.66 times bigger than CV. The test showed that the LC_50_ of AP-PLA2 is 1.638 mg/ml. Antiviral analysis of AP-PLA2 *in vitro* showed the inhibition of HIV infection to PBMC. HIV culture with AP-PLA2 and without AP-PLA2 has shown the number of infected PBMC (0.299±0.212% and 9.718±0.802%). Subsequently, RNA amplification of HIV using reverse transcriptase-polymerase chain reaction resulted in the decrease of band intensity in gag gene of HIV.

**Conclusion:**

This research suggests that AP-PLA2 has the potential to develop as an antiviral agent because *in vitro* experiment showed its ability to decrease HIV infection in PBMC and the number of HIV ribonucleic acid in culture.

## Introduction

The crown-of-thorns starfish *Acanthaster planci* is one of the most dangerous coral predators currently contributing to the degradation and loss of Indonesia’s highly diverse reefs, which is a major problem for coral management programs in the Pacific Ocean [1,2]. Outbreaks of *A. planci* have occurred at many locations throughout the Indo-Pacific region as a result of anthropogenically elevated nutrient levels and overfishing [3,4]. Techniques used to manage these outbreaks include implementing biosecurity measures and managing the environmental conditions that lead to outbreaks and disrupting the starfish spawning success [ 4-6]. These management strategies give rise to large quantities of starfish waste; in the present study, we aim to identify ways in which this waste may be utilized [7-10].

The glandular tissue around the venomous spines on the body surface of *A. planci* produces toxins. Crude venom (CV) extracted from *A. planci* has a range of biological activities including hemolytic, myonecrotic, capillary permeability-increasing, hemorrhagic, edema-forming, mast cell histamine-releasing, phospholipase A2 (PLA2), anticoagulant, and cardiovascular activity, as well as mouse lethality [11]. CV comprises several bioactive protein toxins, including plancinin, plancitoxins I and II, and PLA2 enzymes [8]. Plancinin acts as a coagulant factor in the human blood coagulation cascade through the activation of prothrombin, and it significantly inhibits factor X activation through both intrinsic (factor IXa–factor VIIIa–phospholipids–Ca21) and extrinsic (factor VIIa–tissue factor–phospholipids–Ca21) mechanisms [12]. Plancitoxins have potent hepatotoxicity, similar to that of mam malian deoxyribonuclease II, which results in deoxyribonucleic acid (DNA) degradation during apoptosis and engulfment-mediated DNA degradation [11]. *A. planci* phospholipases A2 (AP-PLA2-I and -II) have hemolytic activity only in the presence of phosphatidylcholine (PC), which releases fatty acids that act as antibacterial agents and also possess myotoxic activity [10,13].

PLA2 can be extracted from the venomous spines of *A. planci* [8,10]. In a previous study, PLA2 was purified from *A. planci* using a rapid and efficient method, which produced a single band of PLA2 protein with specific activity 3-20 times stronger than that of CV [14,15].

PLA2 from snake venom exhibits antiviral activity against human immunodeficiency virus (HIV); it interacts with host cells and prevents the intracellular release of virus capsid proteins, thereby blocking viral entry into the cells before virion uncoating [16,17]. PLA2 from bee venom has been shown to block the replication of both M- and T-tropic HIV virions by behaving as a ligand for the HIV-1 coreceptor CXCR4 [16,18]. AP-PLA2 exhibits some of the same characteristics as PLA2 from snake and bee venom, suggesting that AP-PLA2 could potentially have anti- viral activity against HIV.

Based on this information, the present study explores the characteristics of AP-PLA2 and assesses its potential as a cheap, natural, safe, and environmentally friendly drug for the treatment of HIV infection.

## Materials and Methods

### Ethical approval

Ethical approval to conduct the study was granted by Institute of Human Virology and Cancer Biology, Faculty of Medicine, Universitas Indonesia (No. 0108/SK/R/UI/2010).

### *A. planci* samples

*A. planci* samples used in this experiment were obtained from the Moluccas Islands, eastern Indonesia, in December 2011.

### AP-PLA2 isolation

AP-PLA2 was isolated using the extraction method described by Savitri *et al*. [14,15]. Briefly, 50 g of venomous *A. planci* spines was sonicated in 100 ml 0.01 M phosphate buffer (pH 7.0) and 0.001 M CaCl_2_, and the extract was centrifuged at 15,000 rpm at 4°C for 30 min. The supernatant (CV) was obtained. The *A. planci* CV was heated at 60°C for 30 min and centrifuged at 15,000 rpm at 4°C for 30 min. The supernatant (heated venom) was thus obtained. Isolate of AP-PLA2 was obtained from the heated venom by precipitation with 20% saturated ammonium sulfate and centrifugation at 30,000 rpm at 4°C. Subsequently, precipitated AP-PLA2 was dissolved in 0.01 M pH 7.0 phosphate buffer (K_2_HPO_4_ and KH_2_PO_4_) and 0.001 M CaCl_2_ for storage at 4°C until use.

### AP-PLA2 activity test

Throughout the aforementioned purification procedure, the activity of AP-PLA2 was assessed using a method described by Marinetti based on its enzymatic activity on egg yolk [19].

### Peripheral blood mononuclear cell (PBMC) isolation and culture

PBMCs were isolated from fresh whole blood with Ficoll-Paque. After isolation, PBMCs were stimulated with phytohemagglutinin (PHA) for 3 days before being cultured for 14 days.

### Toxicity test

To obtain toxicity data, 500,000 primary PBMCs were aliquoted and dropped into treatment wells; subsequently, CV and various amounts of F20 (AP-PLA2 with 20% ammonium sulfate; 5, 4, 3, 2, and 1 mg/ml) were added to the culture. PBMCs alone were used as a control. PBMC growth was measured using cytometer on days 0, 4, 8, 12, and 14. From these measurements, the median lethal dose (LC_50_) of AP-PLA2 (the dose of AP-PLA2 that killed exactly 50% of the PBMCs) was calculated.

### HIV culture

HIV culture was obtained by adding HIV-infected PBMCs from an infected patient to normal PHA-stimulated PBMCs. Three cultures were created: PBMC culture as an uninfected control culture, PBMC culture with HIV as a negative control, and PBMC culture with HIV and AP-PLA2 as a test culture. Cultures were incubated for 7 days. Subsequently, RNA was extracted from the cells for reverse transcriptase-polymerase chain reaction (RT-PCR) and immunofluorescence analysis.

### RT-PCR

RT-PCR was performed at the Institute of Human Virology and Cancer Biology, Universitas Indonesia (IHVCB-UI), Jakarta, Indonesia, which is equipped with biosafety level 3 equipment. Before RNA extraction, the PBMC-HIV culture was centrifuged at 1300 rpm for 10 min. Supernatant obtained by centrifugation was collected for further processing. HIV RNA was extracted from the PBMC culture sediment using QIA quick PCR Purification Kit (QIAGEN KIT). The RNA was then amplified using SuperScript™ III Reverse Transcriptase (SS™ III enzyme). Primers used for the first cycle were H1857 and H1967C, which specifically amplify HIV group-specific antigen (Gag) proteins. The thermocycling process was as follows: 55°C for 30 min, 94°C for 2 min, and 35× for the following cycles: 94°C for 15 s, 55°C for 30 s, 68°C for 90 s, and finally 68°C for 5 min.

After amplification, the RNA was subjected to 12% polyacrylamide gel electrophoresis. Electrophoresis run for 50 min at 120 V, and the band was imaged with Gel Doc XR [20].

### Indirect immunofluorescence assay

HIV RNA was fixed in 500 μl, 3.7% formaldehyde for 15 min at 20°C; next, 0.2% Triton X-100 in phosphate-buffered saline was added for permeabilization. Subsequently, 150 μl of fluorescence isothiocyanate (FITC) was added, and the mixture was incubated at 20°C for 1 h. Immunofluorescence assay was performed using real-time flow cytometry quantification equipment using Thermo Fisher Scientific™ green fluorescent protein marker [21].

## Results

### AP-PLA2 activity

AP-PLA2 is an enzyme in the phospholipase group. Its activity can be measured using egg yolk because of its ability to degrade PC: The purer the yolk, the higher the AP-PLA2 activity.

**Figure-1:**
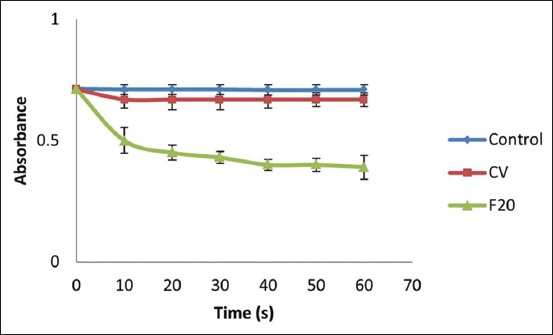
Peripheral blood mononuclear cell treated with both PLA2 of crude venom (CV) and PLA2 with 20% fractioned by ammonium sulfate (F20) shows decreasing absorbance promptly. The final absorbance is control 0.709±0.02 (n=3), CV 0.67±0.02 (n=3), and F20 0.39±0.05 (n=3).

The enzyme activity was measured by a UV-vis spectrophotometer at λ 900 nm. One enzyme unit is estimated as an activity that causes a reduction of 0.01 absorbance/min. As shown in Figure-1, CV exhibited less specific activity compared with others. Meanwhile, F20 exhibited greater specific activity and made the egg yolk 15.7 times more pure than CV did.

### Toxicity test

**Figure-2:**
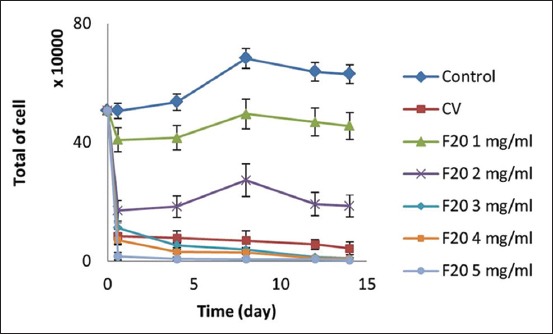
Toxicity test result of PLA2 to peripheral blood mononuclear cell (PBMC) culture. Addition of PLA2 in PBMC culture significantly decreases total of cell. Final total of cell (day 14) is in control 630,000±31,500 (n=3), crude venom 43,750±21,875 (n=3), PLA2 with 20% fractioned by ammonium sulfate (F20) 1 mg/ml 455,000±45500 (n=3), F20 2 mg/ml 186,250±37,250 (n=3), F20 3 mg/ml 10,000±1,500 (n=3), F20 4 mg/ml 7,500±3,750 (n=3), and F20 5 mg/ml 3750±3000 (n=3).

After the addition of 2-5 mg/ml CV and F20 extract (Figure-2), fewer than 300,000 cells remained in the PBMC culture; this indicates that the rate of cell death was very high. Addition of 1 mg/ml F20 resulted in a cell death rate of approximately 8.81%. The lower the concentration of toxin added, the less cell death occurred in the PBMC culture; this can be expressed as y = 5.506x − 12.698. Probit analysis (Table-1) was conducted to determine LC_50_ [22], which was 1.64 mg/ml.

**Table-1:** Probit value, transformation result from percentage of mortality.

[C] (μg/mL)	Log [C]	% mortality	Probit value
0.00	-	0	0
1.00	3.00	8.81	3.64
2.00	3.30	70.27	5.55
3.00	3.47	97.05	6.88
4.00	3.60	98.52	7.15
5.00	3.69	99.01	7.33

### Antiviral activity test

**Figure-3:**
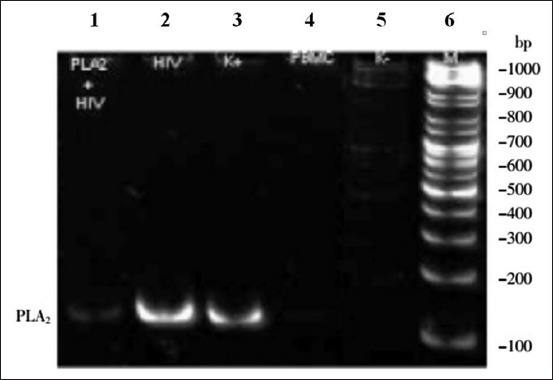
Gel electrophoresis data after reverse transcriptase-polymerase chain reaction human immunodeficiency virus (HIV) ribonucleic acid (RNA) fluorescence. Lane 1: HIV culture was added to PLA2 (PLA2+HIV). Lane 2: HIV culture without treatment (HIV). Lane 3: Cultured line control of a positive HIV RNA (K+). Lane 4: The culture of peripheral blood mononuclear cell. Lane 5: Cultured line control of a negative HIV RNA (K-). Lane 6: Standard protein.

Gel electrophoresis results are shown in Figure-3. These results, taken together with the results of the immunofluorescence assay, indicate that HIV was present in the range of 105 bp in the control HIV culture as well as in the AP-PLA2+HIV culture. From the results of RT-PCR, we can conclude that AP-PLA2 exhibits antiviral activity against HIV. Using gray tape, 1^st^ lane ribbon (HIV culture was added to PLA2; PLA2+HIV) was compared to both of 2^nd^ lane ribbon (HIV culture without treatment; HIV) and 3^rd^ lane ribbon (Cultured line control of a positive HIV RNA; K+).

**Figure-4:**
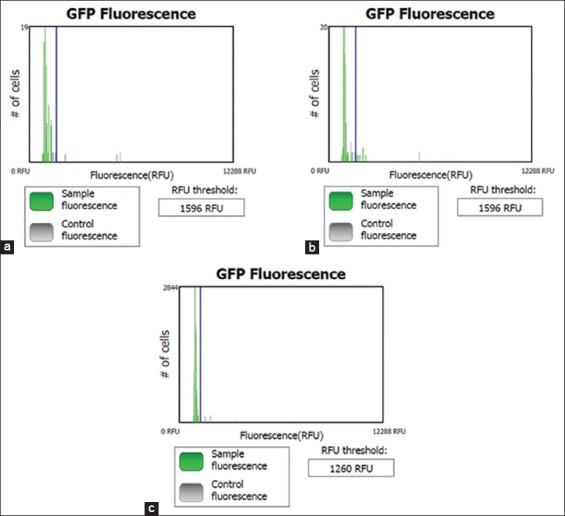
Cytometry of immunofluorescence assay using fluorescence isothiocyanate in (a) peripheral blood mononuclear cell culture as control negative, (b) no treatment human immunodeficiency virus (HIV) culture as a positive control, (c) HIV culture treat by the addition of PLA2 (PLA2+HIV). The peak by green color on the right side of a threshold shows among of expression of HIV protein. The graph indicates that amount of expressed HIV protein in PLA2+HIV culture is less than no treatment HIV culture.

An immunofluorescence assay incorporating FITC cytometry followed by flow cytometry was used to assess the number of HIV-infected cells in the PBMC culture: The greater the fluorescence, the higher the infection rate (Figure-4).

**Figure-5:**
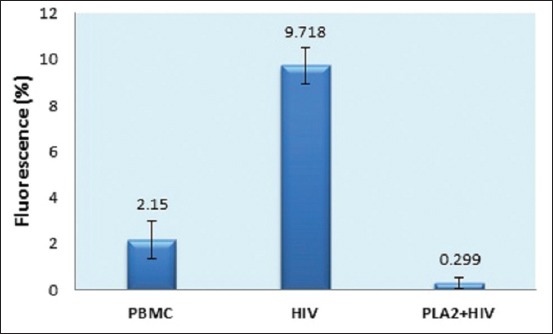
Percentage of human immunodeficiency virus (HIV) infection in peripheral blood mononuclear cell (PBMC) culture line. After addition of PLA2, proportion of HIV infection was decreased significantly from HIV infection without treatment (HIV) about 9.718±0.802% (n=4) to HIV infection with treatment (PLA2+HIV) 0.299±0.212% (n=4). Meanwhile, control was about 2.15±0.802% (n=4).

The untreated HIV culture produced five green peaks, while the AP-PLA2+HIV culture produced only two green peaks. The cell fluorescence in the PBMC, HIV, and AP-PLA2+HIV cultures was approximately 2.16%, 9.72%, and 0.29%, respectively (Figure-5). The amount of HIV-infected cells dropped significantly after the addition of AP-PLA2, that is, from 9.72% in the HIV - only culture to 0.299% in the AP-PLA2+HIV culture.

## Discussion

The antiviral activity of CV is low because it is impure, unlike F20, which has higher AP-PLA2 content and therefore exhibits more significant antiviral activity.

The addition of F20 containing 1.64 mg/ml AP-PLA2 results in approximately 50% PBMC death. This is the LD_50_ or the maximum dosage tolerated by the PBMCs. Therefore, the concentration of PLA2 must be below this value for more than 50% of cells to survive. This is a lower value than the LD_50_ of bee venom for an adult human, which was reported to be 28 mg of venom per kg of body weight. The main component of bee venom is melittin (40%- 50%), and PLA2 is present in much smaller amounts (10%-12%) [18,23].

It has been suggested that two types of nanoparticle encapsulation therapies using melittin are possible: A vaginal gel for preventing the spread of HIV infection and treatment of existing HIV infections, particularly drug-resistant ones [23]. Melittin is effective in inhibiting HIV type 1 (HIV-1) infection, with a 50% inhibitory concentration (IC_50_) of 2.4 µM for CXCR4-tropic viral strains and 3.6 µM for CCR5-tropic viral strains [24].

The HIV cultures were incubated for 7 days to ensure that both PBMCs and HIV grew. Next, the culture was used for qualitative assay by RT-PCR, cytometry, and indirect immunofluorescence assay. RT-PCR showed the anti-HIV activity of AP-PLA2 by detecting HIV RNA. An immunofluorescence assay was also performed to show AP-PLA2 activity by detecting the expression of HIV protein in cells. Both tests were used to assess the antiviral activity of AP-PLA2. The difference in intensity of the PCR bands reflects differences in the expression of RNA; the HIV RNA detected in the PLA2+HIV culture produced a weaker band than that produced by the untreated HIV control culture, which shows that the growth of HIV in the presence of AP-PLA2 was much less than the growth of untreated HIV and indicates that AP-PLA2 has an inhibitory effect on HIV replication.

Qualitative analysis by immunofluorescence cytometry indicated that AP-PLA2 could decrease the amount of HIV protein that was expressed in PBMCs. AP-PLA2 reacts with phospholipids breaking them down into fatty acids and phosphoglycerides; in this way, it lyses the HIV phospholipid bilayer envelope, causing the virus to become inactive.

Taken together, the findings of this study support the hypothesis that AP-PLA2 exhibits antiviral activity that may inhibit HIV replication. Our results are similar to those reported by previous researchers, who concluded that a human-specific secretory PLA2 (sPLA2-X) could neutralize HIV-1 through degradation of the HIV membrane. This catalytic function was required for antiviral activity and did not affect the infected cells. sPLA2-X has been shown to inhibit the replication of tropic HIV-1 CCR and CXCR in human CD4^+^ T cells (T-helper cells, which are white blood cells that play a principal role in protecting the body from infection) and potentially reduce gene transfer among HIV-1 envelope pseudotyped viral vectors. Viral particles resistant to damage by complement factors and antibodies were sensitive to lysis by sPLA2-X. Therefore, sPLA2-X has been suggested as a novel antiviral agent that acts independently of the acquired immune system [25].

## Conclusion

AP-PLA2 has been shown to decrease the HIV infection rate and the HIV-specific RNA concentration in *in vitro* PBMC culture. To conclude, the *A. planc*i crown based on HIV infection in PBMC was significantly around 98%. In the present study, AP-PLA2 was produced from CV by a simple extraction process. Because CV is a waste material and the extraction process is simple, AP-PLA2 is environmentally friendly and cheap. Although further study is required to determine the safety and efficacy of AP-PLA2 *in vivo* throughout the entire human body, this waste product is a promising source of a potential anti-HIV agent that could potentially provide an effective solution not only to the challenges facing coral reef conservation programs but also in treating one of the most dangerous diseases affecting humanity today.

## Author’s Contributions

AW and MS: Arranged, designed, and supervised the study. KL carried out sampling, laboratory analy­sis and wrote the first draft of the manuscript. AW, MS and KL: Analyzed the data. AW, DKP and MS: Contributed to the writing of the manuscript. MS, AW and HH: Jointly developed the structure and arguments for the paper. AW, KL, HH, DKP and MS: Agree with manuscript results and conclusions, also made critical revisions and approved final version. All authors read and approved of the final manuscript.
